# The NHGRI-EBI GWAS Catalog: knowledgebase and deposition resource

**DOI:** 10.1093/nar/gkac1010

**Published:** 2022-11-09

**Authors:** Elliot Sollis, Abayomi Mosaku, Ala Abid, Annalisa Buniello, Maria Cerezo, Laurent Gil, Tudor Groza, Osman Güneş, Peggy Hall, James Hayhurst, Arwa Ibrahim, Yue Ji, Sajo John, Elizabeth Lewis, Jacqueline A L MacArthur, Aoife McMahon, David Osumi-Sutherland, Kalliope Panoutsopoulou, Zoë Pendlington, Santhi Ramachandran, Ray Stefancsik, Jonathan Stewart, Patricia Whetzel, Robert Wilson, Lucia Hindorff, Fiona Cunningham, Samuel A Lambert, Michael Inouye, Helen Parkinson, Laura W Harris

**Affiliations:** European Molecular Biology Laboratory, European Bioinformatics Institute, Wellcome Genome Campus, Hinxton, Cambridge CB10 1SD, UK; European Molecular Biology Laboratory, European Bioinformatics Institute, Wellcome Genome Campus, Hinxton, Cambridge CB10 1SD, UK; European Molecular Biology Laboratory, European Bioinformatics Institute, Wellcome Genome Campus, Hinxton, Cambridge CB10 1SD, UK; Open Targets, Wellcome Genome Campus, Hinxton, Cambridge CB10 1SD, UK; European Molecular Biology Laboratory, European Bioinformatics Institute, Wellcome Genome Campus, Hinxton, Cambridge CB10 1SD, UK; Wellcome Sanger Institute, Wellcome Genome Campus, Hinxton, Cambridge CB10 1SA, UK; Health Data Research UK Cambridge, Wellcome Genome Campus and University of Cambridge, Cambridge, UK; European Molecular Biology Laboratory, European Bioinformatics Institute, Wellcome Genome Campus, Hinxton, Cambridge CB10 1SD, UK; European Molecular Biology Laboratory, European Bioinformatics Institute, Wellcome Genome Campus, Hinxton, Cambridge CB10 1SD, UK; Division of Genomic Medicine, National Human Genome Research Institute, National Institutes of Health, Bethesda, MD 20892, USA; European Molecular Biology Laboratory, European Bioinformatics Institute, Wellcome Genome Campus, Hinxton, Cambridge CB10 1SD, UK; European Molecular Biology Laboratory, European Bioinformatics Institute, Wellcome Genome Campus, Hinxton, Cambridge CB10 1SD, UK; European Molecular Biology Laboratory, European Bioinformatics Institute, Wellcome Genome Campus, Hinxton, Cambridge CB10 1SD, UK; European Molecular Biology Laboratory, European Bioinformatics Institute, Wellcome Genome Campus, Hinxton, Cambridge CB10 1SD, UK; European Molecular Biology Laboratory, European Bioinformatics Institute, Wellcome Genome Campus, Hinxton, Cambridge CB10 1SD, UK; European Molecular Biology Laboratory, European Bioinformatics Institute, Wellcome Genome Campus, Hinxton, Cambridge CB10 1SD, UK; European Molecular Biology Laboratory, European Bioinformatics Institute, Wellcome Genome Campus, Hinxton, Cambridge CB10 1SD, UK; European Molecular Biology Laboratory, European Bioinformatics Institute, Wellcome Genome Campus, Hinxton, Cambridge CB10 1SD, UK; European Molecular Biology Laboratory, European Bioinformatics Institute, Wellcome Genome Campus, Hinxton, Cambridge CB10 1SD, UK; European Molecular Biology Laboratory, European Bioinformatics Institute, Wellcome Genome Campus, Hinxton, Cambridge CB10 1SD, UK; European Molecular Biology Laboratory, European Bioinformatics Institute, Wellcome Genome Campus, Hinxton, Cambridge CB10 1SD, UK; European Molecular Biology Laboratory, European Bioinformatics Institute, Wellcome Genome Campus, Hinxton, Cambridge CB10 1SD, UK; European Molecular Biology Laboratory, European Bioinformatics Institute, Wellcome Genome Campus, Hinxton, Cambridge CB10 1SD, UK; European Molecular Biology Laboratory, European Bioinformatics Institute, Wellcome Genome Campus, Hinxton, Cambridge CB10 1SD, UK; European Molecular Biology Laboratory, European Bioinformatics Institute, Wellcome Genome Campus, Hinxton, Cambridge CB10 1SD, UK; Division of Genomic Medicine, National Human Genome Research Institute, National Institutes of Health, Bethesda, MD 20892, USA; European Molecular Biology Laboratory, European Bioinformatics Institute, Wellcome Genome Campus, Hinxton, Cambridge CB10 1SD, UK; European Molecular Biology Laboratory, European Bioinformatics Institute, Wellcome Genome Campus, Hinxton, Cambridge CB10 1SD, UK; Cambridge Baker Systems Genomics Initiative, Department of Public Health and Primary Care, University of Cambridge, Cambridge, UK; British Heart Foundation Cardiovascular Epidemiology Unit, Department of Public Health and Primary Care, University of Cambridge, Cambridge, UK; Health Data Research UK Cambridge, Wellcome Genome Campus and University of Cambridge, Cambridge, UK; Cambridge Baker Systems Genomics Initiative, Department of Public Health and Primary Care, University of Cambridge, Cambridge, UK; British Heart Foundation Cardiovascular Epidemiology Unit, Department of Public Health and Primary Care, University of Cambridge, Cambridge, UK; Health Data Research UK Cambridge, Wellcome Genome Campus and University of Cambridge, Cambridge, UK; Cambridge Baker Systems Genomics Initiative, Baker Heart and Diabetes Institute, Melbourne, Australia; European Molecular Biology Laboratory, European Bioinformatics Institute, Wellcome Genome Campus, Hinxton, Cambridge CB10 1SD, UK; European Molecular Biology Laboratory, European Bioinformatics Institute, Wellcome Genome Campus, Hinxton, Cambridge CB10 1SD, UK

## Abstract

The NHGRI-EBI GWAS Catalog (www.ebi.ac.uk/gwas) is a FAIR knowledgebase providing detailed, structured, standardised and interoperable genome-wide association study (GWAS) data to >200 000 users per year from academic research, healthcare and industry. The Catalog contains variant-trait associations and supporting metadata for >45 000 published GWAS across >5000 human traits, and >40 000 full *P*-value summary statistics datasets. Content is curated from publications or acquired via author submission of prepublication summary statistics through a new submission portal and validation tool. GWAS data volume has vastly increased in recent years. We have updated our software to meet this scaling challenge and to enable rapid release of submitted summary statistics. The scope of the repository has expanded to include additional data types of high interest to the community, including sequencing-based GWAS, gene-based analyses and copy number variation analyses. Community outreach has increased the number of shared datasets from under-represented traits, e.g. cancer, and we continue to contribute to awareness of the lack of population diversity in GWAS. Interoperability of the Catalog has been enhanced through links to other resources including the Polygenic Score Catalog and the International Mouse Phenotyping Consortium, refinements to GWAS trait annotation, and the development of a standard format for GWAS data.

## INTRODUCTION

The question of how genetic variation in a population influences phenotypic variation and disease risk is of major importance in biology. Genome wide association studies (GWAS) identify associations between hundreds of thousands of genomic variants and complex traits or diseases. GWAS have revolutionised the study of complex disease in the last ten years, providing insights into the genetic architecture of diseases through discovery of novel associations, the identification of disease susceptibility loci and biological pathways enabling biomarker discovery.

The NHGRI-EBI GWAS Catalog (www.ebi.ac.uk/gwas) is the largest and most complete publicly available resource of Findable, Accessible, Interoperable and Reusable (FAIR) GWAS data. The Catalog is highly accessed by users in academic research, healthcare and industry, through a graphical user interface (GUI), downloadable spreadsheets and APIs. In the last year (July 2021–June 2022), there were >200 000 unique website users (in 186 countries) and >2.5 million page views, a ∼90% and ∼70% increase, respectively, on the same period in 2018–2019, when our last database update was published (1). Programmatic access is increasingly popular with ∼30 million API requests in 2021, a 6-fold increase over 2019.

Users employ the structured and richly annotated data provided by the GWAS Catalog for a wide range of applications that require prompt and open access. These include prioritising loci for functional study, drug discovery, predicting disease risk, quantifying genomic diversity, GWAS meta-analysis, fine mapping and causality studies, integration with additional data, and input into analytical tools. Some key resources that ingest our data include Ensembl (https://www.ensembl.org) (2); variant prioritisation applications such as PheGenI (https://www.ncbi.nlm.nih.gov/gap/phegeni) (3), Open Targets Genetics (https://genetics.opentargets.org/) (4) and HuGeAMP Knowledge Portals (https://hugeamp.org/); and other databases and tools such as MRC IEU OpenGWAS (https://gwas.mrcieu.ac.uk/) (Elsworth *et al.*, bioRxiv, https://doi.org/10.1101/2020.08.10.244293), PhenoScanner (http://www.phenoscanner.medschl.cam.ac.uk/) (5,6) and GWAS Central (https://www.gwascentral.org/) (7).

Data is acquired through a combination of deep learning methods to identify publications, curation of publications by expert scientists, and direct data submission by authors. The Catalog's scope covers the most significant variant-trait associations, as well as structured metadata about the publication, study design, samples and traits, for all published human GWAS. Wherever possible, the full *P*-value summary statistics for each study are also collected and made publicly available. The Catalog uses transparent curation and acceptance criteria (https://www.ebi.ac.uk/gwas/docs/methods) which are regularly updated to reflect user needs and changes in the data generated.

## INCREASING DATA VOLUME AND COMPLEXITY

**Figure 1.**
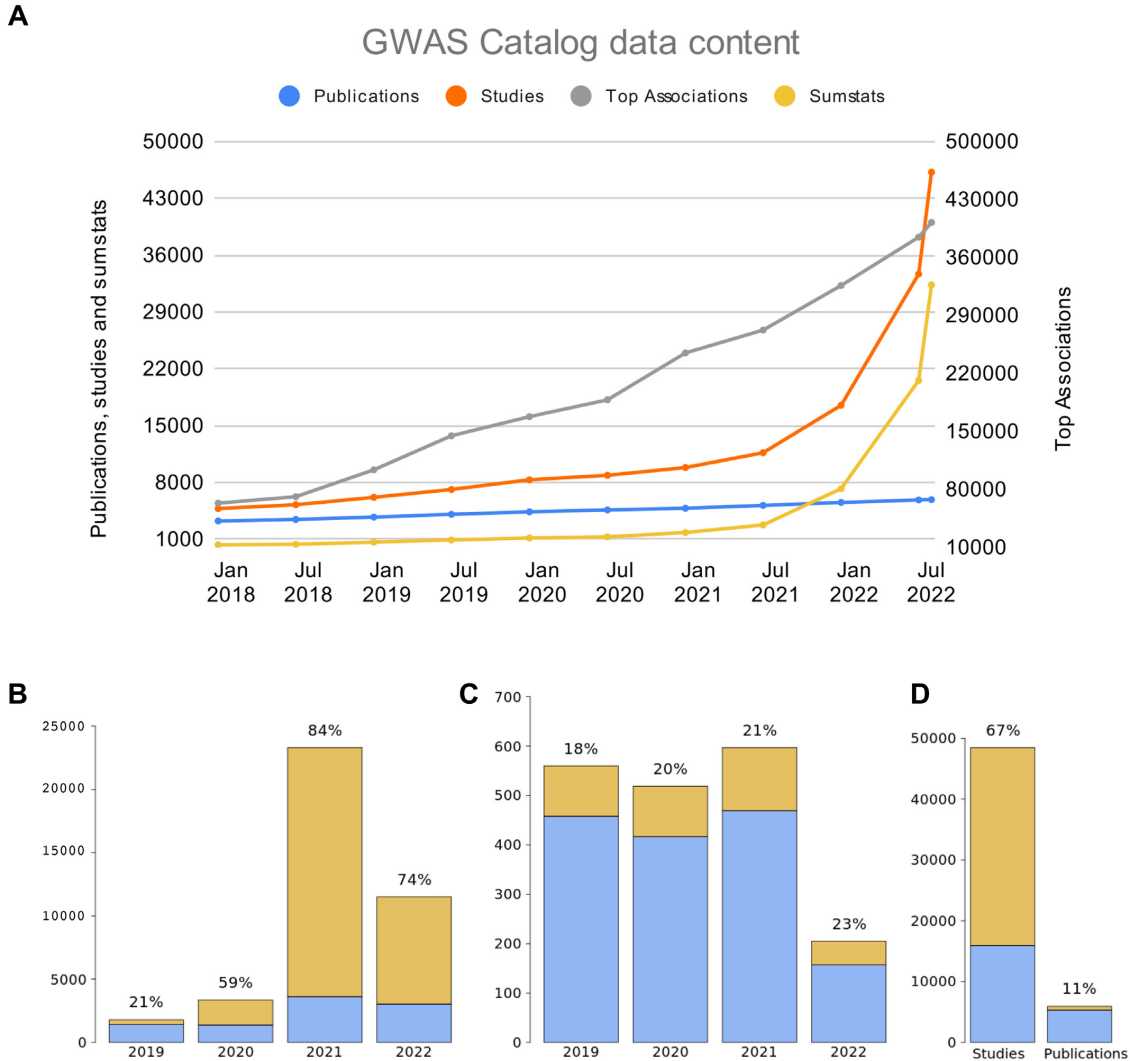
The increase in GWAS Catalog data content since 2018. (**A**) Cumulative numbers of publications, studies, studies with summary statistics, and associations over the period, by publication year. (**B–D**) Summary statistics sharing by publication year for (**B**) GWA studies (**C**) publications and in the Catalog overall (**D**).

The overall volume of GWAS data generation has grown considerably (Figure 1A). As of July 2022, the GWAS Catalog contains ∼400 000 curated SNP-trait associations from >45 000 individual GWAS in ∼6000 publications. Whilst the number of publications has approximately doubled since 2018, the number of GWAS has increased 8-fold, and associations 5-fold. These numbers reflect the growth in large-scale analyses of multiple traits in individual publications, e.g. in fields such as proteomics, metabolomics, and brain imaging. Large cohorts (e.g. national biobanks such as UK Biobank and FinnGen) with data for thousands of diseases and other traits have become a major part of the GWAS landscape. Some publications now contain thousands of distinct GWAS analyses (8,9), and the mean number of GWAS per publication has grown from three in 2018 to 39 in 2021.

Complete GWAS summary statistics provide more detailed information than the lead associations alone, allowing greater opportunities for downstream analysis and reuse, while maintaining participant privacy. The GWAS Catalog is now one of the largest, most visited and most frequently updated resources of freely available GWAS summary statistics. The repository includes >40 000 datasets from >600 peer-reviewed publications and >3500 traits (Figure 1B–D). Users can access and download summary statistics from the GWAS Catalog FTP site in the original format and harmonised to the most recent genome assembly for interoperability (https://www.ebi.ac.uk/gwas/docs/methods/summary-statistics) (1). The increasing trend to share these datasets is reflected in the huge increases in availability of summary statistics in the GWAS Catalog over time (Figure 1A).

Changing data generation technology has meant that whole exome (WES) and whole genome (WGS) sequencing data are now available for millions of individuals, including large GWAS cohorts such as UK Biobank and TOPMed. The Catalog has therefore expanded its scope to include sequencing-based GWAS (seqGWAS) data (10) and began routinely curating single-variant seqGWAS published from 2021 onwards. To date, >5000 seqGWAS from >50 publications have been included in the Catalog (14% of studies and 5% of publications curated between Q1 2021 and Q2 2022), including >4000 summary statistics. Statistical methods that evaluate aggregate association over multiple variants in a gene or genomic region are commonly used for rare-variant association testing. Due to high user demand, we are making summary statistics available from such analyses, with >3900 genebased GWAS included to date (8). Novel methods for genome wide analysis of copy number variation (CNV) have recently been applied to large datasets revealing previously undiscovered associations. Following user request, we have made these summary statistics available in the Catalog (250 studies from two publications) (11,12). With input from the community, we are working towards making associations from both gene-based and CNV analyses searchable in the Catalog.

As the scale and complexity of GWAS data continues to grow, the user community requires rapid access to datasets with larger sample sizes, more extensive genotyping, more traits per publication and full *P*-value summary statistics to make optimal use of GWAS results for downstream applications. However, increased scale also creates challenges for data ingestion, storage and access. The GWAS Catalog has responded to these challenges by (a) acquiring author-submitted content, (b) developing new workflows and infrastructure processes to increase throughput and (c) prioritising inclusion of data that will add the most value to the resource. These improvements have ensured that we continue to make essential data available to users within a reasonable timeframe as content scales up.

## AUTHOR-SUBMISSION SYSTEM

**Figure 2.**
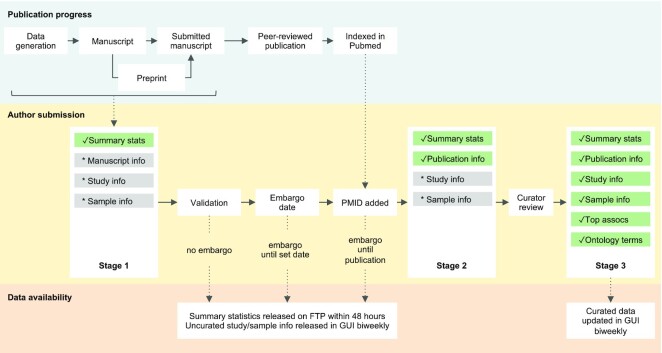
The submission and review process for unpublished data submissions. This workflow covers the majority of submissions to date (74%), and shows the progress from uncurated submitted data (*), to validated/curated data (✓). Authors submit at any point before journal publication, by providing summary statistics and supporting metadata (Stage 1), and receive a persistent identifier for each study. Summary statistics are validated upon submission. Metadata may be made available upon submission, or embargoed by the author until journal publication or a specific date. Final publication details are confirmed once an article is indexed in PubMed (Stage 2). Study and sample information are confirmed and updated after review by a curator, who also adds top associations, ontology annotations and any replication information for each study (Stage 3). Curated data are released biweekly.

A major recent development has been the implementation of a new submission system, built on FAIR principles, to allow authors to directly submit GWAS summary statistics to the Catalog. Journals increasingly recommend or require authors to submit their summary statistics prior to publication, therefore submissions can be made at any point prior to publication in a journal, as well as for existing publications. This not only enables early access to summary statistics for reviewers and other interested parties, but can also fast-track the curation process once papers are published. As of July 2022, we have received 315 submissions comprising >30 000 GWAS, including 232 submissions (74%) for unpublished data, making this the major route for data submission (Figure 2).

The submission portal is accessible from the GWAS Catalog website, at https://www.ebi.ac.uk/gwas/deposition, with a free user account managed by the European Life Sciences Research Infrastructures (Life Science RI). Detailed documentation (https://www.ebi.ac.uk/gwas/docs/submission) and a video walkthrough (https://doi.org/10.6019/TOL.GWAS_catalog-w.2021.00001.1) are provided to assist authors with the submission process.

Authors may submit summary statistics associated with an existing publication (with or without a PMID), accepted or submitted manuscript, pre-print, draft manuscript in preparation, or even a dataset that is not intended for journal publication. This flexibility allows for the submission of GWAS data from varied sources, such as datasets from industry, where journal publication may not be a high priority (e.g. GCST90002217-20, Taylor *et al.*, medRxiv: https://doi.org/10.1101/2020.06.17.20134015). For data that is not yet published in a journal, key information such as author names, titles and identifiers (e.g. preprint DOIs) are collected to ensure provenance and to enable cross-reference of the submission to a potential future publication.

Summary statistics files are transferred to the GWAS Catalog using Globus (13,14). To ensure data completeness, interoperability and reusability, summary statistics are required to conform to the GWAS Catalog's standard format, which includes a consistent .tsv file format, and mandatory and recommended fields (Hayhurst *et al.*, bioRxiv, https://doi.org/10.1101/2022.07.15.500230). A Python-based validation tool, ss-validate (https://pypi.org/project/ss-validate/), is provided for authors to check that their files meet validation criteria before submitting. In order to promote reusability for the widest range of purposes, summary statistics submissions since March 2021 use a CC0 (https://creativecommons.org/publicdomain/zero/1.0/) licence by default, placing them as completely as possible in the public domain. Summary statistics from older submissions, and those acquired by GWAS Catalog curators from external sources, are provided under the EMBL-EBI Terms of Use (https://www.ebi.ac.uk/about/terms-of-use). A small number of datasets are made available under a CC-BY-NC-4.0 licence (https://creativecommons.org/licenses/by-nc/4.0/; restricting commercial use), applied in exceptional circumstances where the submitter is limited by study participant consent and in consultation with the GWAS Catalog.

**Figure 3.**
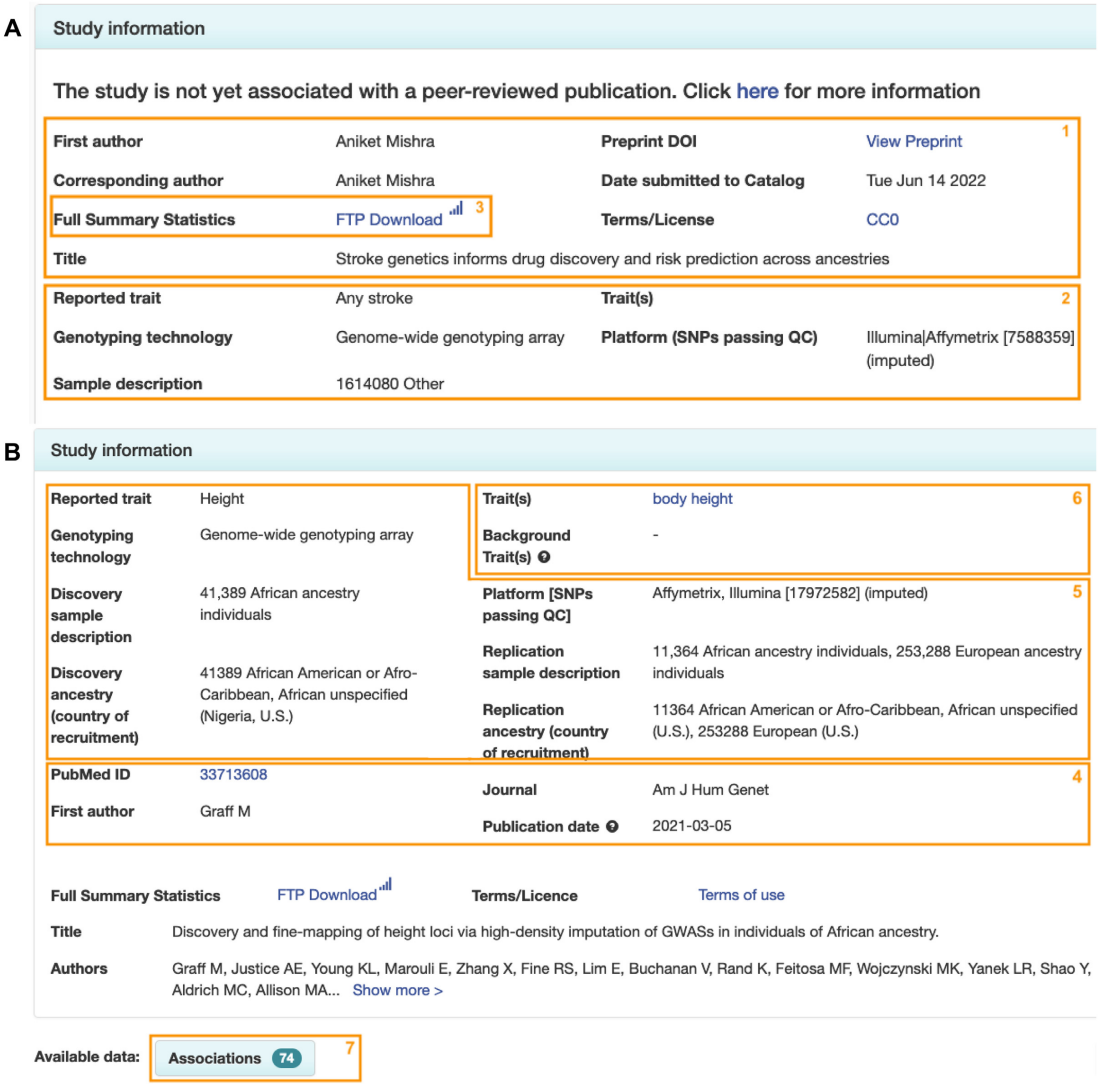
Example screenshots of study landing pages in the GWAS Catalog GUI. (**A**) The landing page for an unpublished study shows provisional publication information such as author names, titles and links to preprints [1]; basic study and sample information [2]; and a link to the full summary statistics [3]. (**B**) The landing page for a published study additionally provides the final journal citation [4], more detailed study and sample information [5], ontology annotations [6] and curated top associations [7].

Authors submit supporting metadata in a spreadsheet. For unpublished submissions, they receive a blank template and provide study design, trait and sample information. Uploaded templates are validated to ensure metadata is consistent and correctly formatted. Upon successful submission, authors receive a list of persistent study accessions, which can be cited in their article, or shared with journals, collaborators and others. Summary statistics are made available on the GWAS Catalog FTP within 48 hours, or at the end of the embargo period, and supporting metadata for each study is displayed at the next biweekly data release, on a dedicated searchable landing page in the Catalog GUI (Figure 3A). FTP directories are named using the study accession assigned at submission, supporting persistent file paths to ensure that users can always access the data in the same way before and after journal publication.

Unpublished submissions are cross-referenced with weekly literature search results to identify any that now have an associated peer-reviewed publication indexed in PubMed. This is done using fuzzy string matching between submission details and published citations, as well as the EuropePMC Article Status Monitor (https://europepmc.org/ArticleStatusMonitor) and direct notification by authors. Once a publication is identified, the submission is updated by the Catalog to include the citation, top associations and ontology mapping (Figure 3B), and additional harmonised versions of the summary statistics are made available via FTP. The summary statistics harmonisation pipeline has been parallelised and optimised, increasing the speed of processing 4-fold, and enabling large datasets to be harmonised and released typically within a few days of inclusion of the publication in the Catalog.

For data already associated with a PubMed-indexed publication at the time of submission, authors are informed about the current status of the publication in the GWAS Catalog and the appropriate submission type is created. Where a publication has been curated and the study and sample information is already available in the Catalog, authors are requested to submit summary statistics only, and to match the files to the corresponding curated studies. Where a publication has not yet been curated, authors are requested to submit both summary statistics and study metadata (as for unpublished submissions above). This allows us to recruit FAIR data of most use to the community.

## SCALING AND VALIDATION OF DATA FLOW

Ingest of author submissions requires an efficient curation workflow to annotate and release submissions every two weeks. In 2022, data releases contained on average 40% more publications, 3000% more studies and 75% more associations than in 2020. Curation system enhancements also improved data representation for publications with many studies. Every new GWAS is now represented accurately as a separate GWAS Catalog study with study-specific trait representation, removing the requirement for *P*-value annotation. Selected older studies have been recurated under the new system, e.g. (15).

We continue to curate top associations and metadata from the literature for additional publications without author submissions. We aim to curate all publications in scope, however prioritisation is applied to ensure data with the highest utility is released first, including publications with significant top SNP/trait associations, full summary statistics available, and those that increase representation of non-European ancestries.

Technical improvements to the Catalog's infrastructure have enabled data to be served at scale. The data release process has been optimised to account for the increased number of studies and associations, including redesign of the genomic mapping pipeline for parallelization, optimised database querying and modular architecture with in-memory computing mechanism for efficient external resource interaction. The pipeline now runs in <10% of the previously required time resulting in timely access to the latest mapping information required for data release. Vertical scaling of the Catalog website and API to cater for increased user requests has been achieved by introduction of new hardware infrastructure and doubling of system resources (memory and computing power) for increased resilience. The latest version of the infrastructure has also been redesigned to use OS-level virtualization and deliver GWAS Catalog software in packages (called containers) orchestrated by Kubernetes which automatically scales the applications horizontally as workload changes, capable of handling spikes, with self-healing capabilities to ensure resilience and maximum uptime.

## TRAIT ANNOTATION IMPROVEMENTS

**Figure 4.**
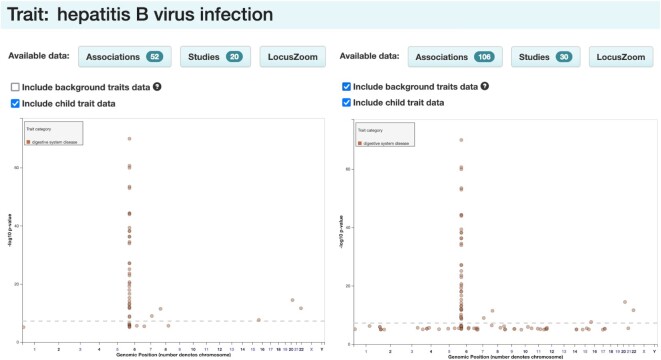
Background trait filter in GUI. When viewing a trait (e.g. hepatitis B virus infection), users can choose whether to display only direct associations with hepatitis B virus infection (left panel, default view), or to additionally display associations with other traits in a background of hepatitis B virus infection (e.g. ‘Liver disease in chronic hepatitis B virus infection’) (right panel). Toggling the filter updates the count of associations and studies for the trait (upper), as well as the plots of associations for the trait across the genome (lower).

Traits are described in a flexible free text field reflecting author language and study design, and annotated using terms from the Experimental Factor Ontology (EFO) (16) to enable searchability and interoperability. More than 5000 diverse EFO terms represent diseases, biomarkers, molecular measurements (e.g. protein QTLs), drug responses and anthropometric measurements. GWA study designs can involve complex relationships between the traits under investigation, which are difficult to represent accurately in a single data field. For example, while the majority of studies (91%) analyse a single trait (e.g. type II diabetes), a significant minority (6%) involve two or more traits related by pleiotropy (e.g. ‘Schizophrenia or bipolar disorder’) or comorbidity/co-occurrence (e.g. ‘Major depressive disorder and alcohol dependence’). Others (3%) involve one or more main traits of interest within the context of an additional background trait—one shared by all participants in the study but not directly tested in the association analysis (e.g. ‘Allergic rhinitis in asthma’). To represent these studies more accurately, we have added a separate field for background trait annotations, leaving only those traits that are directly analysed in the main trait field. Filters have been implemented on each trait page in the GUI to allow users to include or exclude studies where that trait is a background trait (Figure 4). These filters apply to both tabular data and visualisations. We also continue to work with EFO editors to improve the overall structure of the ontology and ensure that it meets the needs of the Catalog's users.

## IMPROVED INTEGRATION WITH OTHER RESOURCES

Development of polygenic scores is an impactful downstream application of GWAS data, that uses summary statistics to make individual-level predictions of genetic predisposition to heritable traits. The Polygenic Score (PGS) Catalog (https://www.pgscatalog.org) was launched in 2019 in collaboration with the GWAS Catalog as an open resource to enable FAIR PGS data and systematic evaluation of published PGS (17). Reciprocal links between the two resources allow users to easily identify polygenic scores that have been generated using data in the GWAS Catalog, and conversely to identify the GWAS source data that was used to develop a given polygenic score. GWAS Catalog studies are linked to PGS by means of a clickable button for relevant study, publication and trait pages, and each score in the PGS Catalog links to the source data used to develop the score in the GWAS Catalog UI. Metadata (e.g. ancestry descriptions) and trait annotations are also harmonised between the two resources.

Additionally, each gene in the GWAS Catalog links to relevant data in the Open Targets drug discovery platform (https://platform.opentargets.org/) and to its mouse ortholog in the International Mouse Phenotyping Consortium (IMPC; https://www.mousephenotype.org). Orthology predictions are provided by IMPC’s reference database, which is rebuilt every week to include the latest HCOP ortholog relationships and data from MGI (18). Such links enhance users’ ability to prioritise candidate variants by integrating additional functional evidence.

## PROMOTING EQUITABLE DATA SHARING

Despite improvements in open data sharing, in 2022, 77% of publications did not share GWAS summary statistics freely at the time of publication (Figure 1B). The GWAS Catalog has addressed barriers to data sharing and re-use via community outreach, noting that barriers are ethical, legal and social as well as technical. In 2020–2021, we analysed the GWAS data landscape through a series of workshops examining FAIR criteria, infrastructure needs and the incentivisation of data sharing (https://www.ebi.ac.uk/gwas/docs/sharing-standardsworkshop) (19).

Analysis performed in March 2021 showed the rate of data sharing differed among different cohorts, with the lowest submission rate in cancer genetics. The proportion of cancer studies that shared sumstats was 4.5× lower than studies of epilepsy (the most widely shared disease trait at the time) and 3× lower than diabetes. A survey revealed the primary reason was data embargoed for use in future research, followed by privacy/ethics issues and lack of awareness of an appropriate data repository. Given the importance of cancer research to human health, we pursued dedicated outreach to the cancer community, achieving a sharp increase in sharing. For publications in 2022, the rate of sharing in cancer is greatly improved with a similar proportion of cancer GWAS sharing summary statistics as for other major disease traits.

Lack of diverse ancestries in genomics studies limits the ability to apply their findings to diverse populations (20). Benefits from improved knowledge of SNP-trait associations accruing from GWAS and PGS are diluted, leading to health inequities for under-represented populations (20,21). The GWAS Catalog has greatly improved the understanding of genomics diversity by providing data necessary to calculate the European bias in published GWAS (20,22). For example, the GWAS Diversity Monitor (https://gwasdiversitymonitor.com) (23) uses the GWAS Catalog as its sole data source. We have observed that the bias towards European ancestry is more pronounced in shared GWAS summary statistics, with 93% of sumstats generated from European-only samples, compared to 79% European-only studies in the Catalog overall.

Resources such as the GWAS Catalog are data gatekeepers and as such have a responsibility to promote data diversity and to review processes to ensure that they do not inadvertently act to reduce the diversity of shared data. In 2021 we convened a working group to explore these issues and identified opportunities to maximise submission of GWAS datasets for diverse populations, which the GWAS Catalog will act on.

These included (a) improved top-down awareness of the benefits of open sharing among patients, cohorts, consortia, researchers, journals and resources; (b) improved messaging around responsibilities of data consumers, with an expectation that analyses of shared data should themselves be shared; (c) appropriate visibility for data generators, to allow less well funded researchers to demonstrate impact; (d) support for under-resourced groups to share data via provision of tools and recruitment of help from the community; (e) adaptable data sharing requirements to mitigate privacy issues and re-identification risk, which can be a barrier to sharing especially in founder populations and (f) promoting full data sharing from multi-ethnic cohorts. The UK Biobank is an example of an extremely successful and well-used multi-ethnic cohort (accounting for at least 13 000 studies in the GWAS Catalog as of July 2022), from which non-European samples are regularly excluded (∼90% of UKB studies in the GWAS Catalog are European-only). This is despite the not insubstantial size of those groups compared to other GWAS sample sets, e.g. ∼8200 self-reported ethnicity as ‘Indian’/‘Pakistani’/‘Bangladeshi’ and ∼8200 self-reported as ‘Black or Black British’ in UKB (https://biobank.ndph.ox.ac.uk/showcase/field.cgi?id=21000), compared to average discovery stage sample size in the GWAS Catalog for ‘South Asian’ (∼6500) and ‘African unspecified’ (∼8500). To minimise exclusions and to facilitate downstream analyses (e.g. GWAS meta-analysis), we encourage data generators to share these data in the GWAS Catalog to enable re-use of the data even when not included in a publication.

## DISCUSSION

The GWAS Catalog has provided FAIR GWAS data to the genomics community for almost 15 years. In the last 3 years, we have migrated from being a fully curated resource to include directly submitted GWAS summary statistics and metadata. This ensures rapid access to full genome-wide datasets, and allows authors to make data available prior to or at the time of publication, fulfilling journal requirements for data sharing. Data is made available with persistent accession IDs and under a clear licence allowing re-use, as recommended by journals and funders, maximising the utility and translational value of the data to the genomics community. The GWAS Catalog is now the recommended GWAS data repository for UK Biobank (https://biobank.ndph.ox.ac.uk/showcase/exinfo.cgi?src=returning_results) and Nature journals (https://www.nature.com/sdata/policies/repositories). Submitted metadata are validated and subject to mandatory field requirements. This improves data completeness over curation from the literature.

A major change in the field in recent years has been the availability of large whole-genome or whole exome sequenced cohorts (e.g. UK Biobank, TOPMed) for GWAS analysis (seqGWAS). We have responded to this change by expanding our scope to include single variant seqGWAS datasets which can be represented in the same way as array GWAS (10) and are now routinely curated in the Catalog. A further challenge is the inclusion of aggregate analyses e.g. gene-based analyses. These are of great interest to the community and provide a valuable link from variant data to functional pathways and drug targets. At the time of our previous publication (10), there were no full *P* value summary statistics from aggregate analyses openly shared to our knowledge. We now accept submissions of these, and are working towards integration of the top associations into the database. Author submission of summary statistics gives us the flexibility to make a wider range of data types available, including aggregate rare variant analyses and CNV analyses, for which a standardised representation of associations or metadata has not yet been defined. Generating a critical mass of these data in a central repository is a valuable first step towards standardisation, interoperability and reusability. We encourage the community to share these data and engage in discussion on a minimal data format. Users are encouraged to always contact the submission helpdesk (gwas-subs@ebi.ac.uk) prior to attempted submission of non-standard genome-wide datasets in order to discuss their suitability.

The success of GWAS and speed at which data can be generated from deeply phenotyped cohorts continues to accelerate. Data sharing initiatives and availability of a central repository for GWAS summary statistics means that large datasets can be shared rapidly, enabling a wide variety of downstream applications and amplifying the downstream knowledge (and cost-effectiveness) gained from any individual study. The GWAS Catalog has met this challenge by developing deposition infrastructure that allows datasets to be shared directly and immediately, lessening the requirement for manual curation. However, this does not remove the scaling issue but rather shifts it towards one of storage and indexing. Our current summary statistics database technology is not performant for cross study queries and we will adopt an alternative strategy to best meet user requirements for querying summary statistics.

We note the landscape of data sharing has changed as research data are generated in healthcare settings, and as federation of data and analyses becomes the norm. Data access models, formats, standards and infrastructure must therefore adapt to new federated models of access. Where data cannot be included in the GWAS Catalog due to ethical or license constraints, it is linked from the Catalog to promote findability. We continue to work with data owners on indexing and standardisation of these data by sharing metadata format specifications, QC and harmonisation tools with the aim of ensuring federated data are also FAIR. Our working groups will continue to address challenges identified to benefit the user community.

## DATA AVAILABILITY

The GWAS Catalog is an open-source project and code is available in the project's github repository (https://github.com/EBISPOT/goci). Curated data are available from the query interface (https://www.ebi.ac.uk/gwas/) and download files from https://www.ebi.ac.uk/gwas/downloads. APIs for the summary statistics data (http://www.ebi.ac.uk/gwas/summary-statistics/api/) and the curated data (https://www.ebi.ac.uk/gwas/docs/programmatic-access) provide programmatic access to all the Catalog's data. The Catalog's GUI also provides access to summary statistics https://www.ebi.ac.uk/gwas/downloads/summary-statistics.

GWAS summary statistics submitted after March 2021 are made available under CC0 terms (https://creativecommons.org/publicdomain/zero/1.0/), while those submitted prior to March 2021 are made available under the standard terms of use for EBI services (https://www.ebi.ac.uk/about/terms-of-use/). We advise consumers of data hosted by the GWAS Catalog to note the licence terms of individual datasets. Other GWAS Catalog data is covered by the EBI terms of use. Code is available under the Apache version 2.0 licence (http://www.apache.org/licenses/LICENSE-2.0).
